# The *dppBCDF *gene cluster of *Haemophilus influenzae*: Role in heme utilization

**DOI:** 10.1186/1756-0500-2-166

**Published:** 2009-08-24

**Authors:** Daniel J Morton, Thomas W Seale, Timothy M VanWagoner, Paul W Whitby, Terrence L Stull

**Affiliations:** 1 Department of Pediatrics, University of Oklahoma Health Sciences Center, Oklahoma City, OK, 73104, USA; 2 Department of Microbiology and Immunology, University of Oklahoma Health Sciences Center, Oklahoma City, OK, 73104, USA; 3 Department of Biology, Oklahoma Christian University, Oklahoma City, OK 73136, USA

## Abstract

**Background:**

*Haemophilus influenzae *requires a porphyrin source for aerobic growth and possesses multiple mechanisms to obtain this essential nutrient. This porphyrin requirement may be satisfied by either heme alone, or protoporphyrin IX in the presence of an iron source. One protein involved in heme acquisition by *H. influenzae *is the periplasmic heme binding protein HbpA. HbpA exhibits significant homology to the dipeptide and heme binding protein DppA of *Escherichia coli*. DppA is a component of the DppABCDF peptide-heme permease of *E. coli*. *H. influenzae *homologs of *dppBCDF *are located in the genome at a point distant from *hbpA*. The object of this study was to investigate the potential role of the *H. influenzae dppBCDF *locus in heme utilization.

**Findings:**

An insertional mutation in *dppC *was constructed and the impact of the mutation on the utilization of both free heme and various proteinaceous heme sources as well as utilization of protoporphyrin IX was determined in growth curve studies. The *dppC *insertion mutant strain was significantly impacted in utilization of all tested heme sources and protoporphyin IX. Complementation of the *dppC *mutation with an intact *dppCBDF *gene cluster *in trans *corrected the growth defects seen in the *dppC *mutant strain.

**Conclusion:**

The *dppCBDF *gene cluster constitutes part of the periplasmic heme-acquisition systems *of H. influenzae*.

## Background

*Haemophilus influenzae *are fastidious facultatively anaerobic Gram-negative bacteria that cause a range of human infections including otitis media, meningitis, epiglottitis and pneumonia [1,2]. *H. influenzae *lacks all enzymes in the biosynthetic pathway for the porphyrin ring and as a result is unable to synthesize protoporphyrin IX (PPIX), the immediate precursor of heme. Since *H. influenzae *cannot synthesize PPIX the organism has an absolute growth requirement for an exogenous source of PPIX or heme [3,4]. As a result of this growth requirement *H. influenzae *has evolved a complex multifunctional array of uptake mechanisms to ensure that it is able to utilize available porphyrin *in vivo *[5]. One protein shown to be involved in utilization of heme by *H. influenzae *is the heme binding lipoprotein HbpA [6-8]. HbpA was initially identified as a potential constituent of a heme acquisition pathway following transformation of an *H. influenzae *genomic DNA library into *Escherichia coli *and screening for recombinant clones with heme-binding activity [7]. Expression of heme-binding activity by *E. coli *correlated with the expression of a protein of approximately 51-kDa, sized on SDS-PAGE gels, that was subsequently purified in a heme-agarose affinity purification protocol, from both recombinant *E. coli *and *H. influenzae*, and shown to be a lipoprotein [7]. Additionally HbpA was localized to the periplasmic space and shown to be associated with both the inner membrane and the outer membrane in *H. influenzae *[7,8]. Subsequently HbpA was shown definitively to have a role in heme utilization in multiple *H. influenzae *strains and to be important in virulence in both mouse and rat models of *H. influenzae *bacteraemia [6,9,10].

HbpA exhibits significant homology to the periplasmic dipeptide binding protein DppA of *Escherichia coli *(for example in comparing HbpA from nontypeable *H. influenzae *strain HI1388 [Genbank Accession No. AAY87900] and DppA from *E coli *K12 substrain MG1655 [Genbank Accession No. AAC76569] the two proteins exhibit 51.3% identity and 64.1% consensus as determined using the AlignX tool of Vector NTI 10.3.0). In *E. coli *DppA functions with the dipeptide ABC transporter DppBCDF to transport both peptides and heme across the periplasmic space [11-13]. This *E. coli *DppABCDF peptide-heme permease is encoded by the consecutive genes *dppABCDF*. In *H. influenzae *the gene encoding HbpA is not located near the genes encoding the *H. influenzae *DppBCDF proteins. In the *H. influenzae *strain Rd KW20 genomic sequence *hbpA *has the locus tag HI0853 while the *dppBCDF *homologs are located at HI1184-1187 [14]. Although it has not been experimentally established bioinformatic analyses indicate that there is a promoter upstream of *dppB *and that the *dppBCDF *gene cluster in *H. influenzae *is transcribed on a polycistronic message. The *H. influenzae *DppBCDF proteins exhibit significant homology to the DppBCDF proteins of *E. coli*; in pairwise comparisons of the proteins from *H. influenzae *strain Rd KW20 and from *E. coli *K12 substrain MG1655 identities were respectively 59.6% for DppB, 61.3% for DppC, 73.1% for DppD and 74.6% for DppF. Since the *dppBCDF *locus in *E. coli *is known to be involved in heme utilization we examined the potential role of the homologous *H. influenzae *locus in the utilization of this essential growth factor.

## Methods

### Bacterial strains and growth conditions

*H. influenzae *Rd KW20 (ATCC 51907) is the strain used in the original *H. influenzae *genome sequencing project and was obtained from the ATCC. *H. influenzae *were routinely maintained on chocolate agar with bacitracin (BBL, Becton-Dickinson, Sparks, MD, USA) at 37°C. When necessary, *H. influenzae *were grown on brain heart infusion (BHI) agar (Difco, Becton-Dickinson, Sparks, MD, USA) supplemented with 10 μg ml^-1 ^heme and 10 μg ml^-1^β-NAD (supplemented BHI; sBHI) and the appropriate antibiotic(s). Heme-deplete growth was performed in BHI broth supplemented with 10 μg ml^-1^β-NAD alone (heme-deplete BHI; hdBHI). Kanamycin was used at 20 μg ml^-1 ^and chloramphenicol was used at 1.5 μg ml^- ^for growth of *H. influenzae*.

### Heme sources

Human hemoglobin, human haptoglobin from pooled human sera, human serum albumin (HSA), and heme (as hemin) and PPIX were purchased from Sigma. Stock heme solutions (1 mg ml^-1 ^heme in 4% v/v triethanolamine) were prepared as previously described [15] (heme is correctly defined as ferrous PPIX while hemin is ferric PPIX; however for the purposes of this manuscript heme is used as a general term and does not indicate a particular valence state). PPIX stock solutions at 1 mg ml^-1 ^were made in water and autoclaved prior to use. Hemoglobin was dissolved in water immediately before use. Hemoglobin-haptoglobin complexes and heme-albumin complexes were prepared as previously described [16,17].

### Construction of a *dppC *insertional mutant

An insertional mutation in *dppC *was constructed as part of an unrelated study [18]. A chromosomal library of *H. influenzae *strain Rd KW20 was constructed as follows: *H. influenzae *chromosomal DNA was digested with *Pvu *II and phosphorylated *Asc *I linkers were ligated to the digested DNA at 15°C overnight. Fragments were separated by agarose gel electrophoresis and fragments in excess of approximately 2000-bp were purified. The purified fragments were digested with *Asc *I and ligated to *Asc *I digested pASC15 (pASC15 is a minimalized vector containing a unique *Asc *I site that was constructed as part of the previous unrelated study [18]). The ligation mixture was transformed into electrocompotent *E. coli *DH5α and recombinant plasmids were recovered. The recombinant *H. influenzae *library was mutagenized using the EZ::Tn<KAN-2> kit (Epicentre technologies) as directed by the manufacturer. Transposon insertion sites were mapped by sequencing out from the transposon unit into the flanking DNA. A plasmid was identified with a transposon insertion within the coding sequence of *dppC *disrupting codon 290 out of a total of 295 codons. The mutated plasmid was designated pASC1262 and was used to transform *H. influenzae *Rd KW20 to kanamycin resistance using a modification of the static-aerobic method as previously described [19]. A kanamycin-resistant transformant with the correct chromosomal rearrangement was identified using the PCR and designated as *H. influenzae *strain TMV1262.

### Complementation of the *dppC *insertional mutant

To complement the *dppC *mutation a plasmid was constructed carrying the entire *dppBCDF operon*. A 4100-bp PCR product, encompassing the entire *dppBCDF *gene cluster, as well as 100-bp upstream of the start codon of *dppB *and 110-bp downstream of the stop codon of *dppF*, was amplified from *H. influenzae *strain Rd KW20 chromosomal DNA using the primers dppBCDF-1 and dppBCDF-2 having the sequences 5'-GGATCCTCCGATAGGATCTGTG-3' and 5'-GGATCCGTGCGGTAGAATTCAAGAG-3' respectively. The primers dppBCDF-1 and dppBCDF-2 were designed to add *Bam *HI sites to each end of the PCR product in order to facilitate subsequent subcloning. The PCR was performed in a 50 μl volume using 100 ng of *H. influenzae *Rd KW20 chromosomal DNA as template, and the reactions contained 2 mM MgCl_2_, 200 μM of each deoxynucleoside triphosphate (New England Biolabs), 10 pmol of each primer and 2 U of Taq DNA Ploymerase (Roche). PCR was carried out for 30 cycles, with each cycle consisting of denaturation at 95°C for 1 min, annealing for 1 min at 56°C and primer extension at 72°C for 4 min with one final extension of 30 min. An amplicon of the expected size was cloned into pCR2.1-TOPO to yield pMB26 and confirmed by automated DNA sequencing. pMB26 was digested with *Bam *HI and the band corresponding to the chromosomally derived insert was ligated to *Bam *HI digested pACYC184, a shuttle vector with the p15a origin of replication that allows establishment of the plasmid in *H. influenzae*, to yield pDJM137. pDJM137 was confirmed by automated DNA sequencing, and was electroporated into the *H. influenzae dppC *mutant strain to yield the corresponding merodiploid strain. Electoporation of *H. influenzae *was carried out as previously described [20] and transformants selected on chloramphenicol. A transformant containing pDJM137 was identified and designated HI2208.

### Growth studies

Growth studies were performed using the Bioscreen C Microbiology Reader (Oy Growth Curves AB Ltd., Helsinki, Finland) as previously described [6,21].

### Statistics

Statistical comparisons of growth between strains under the same growth conditions *in vitro *were made using the Kruskal-Wallis test. Some analyses were made over selected periods of growth as specified in the results. Analyses were performed using Analyse-It for Microsoft Excel v1.71 (Analyze-It Software Inc., Leeds, England). A *P *value < 0.05 was taken as statistically significant.

## Results and discussion

**Figure 1:**
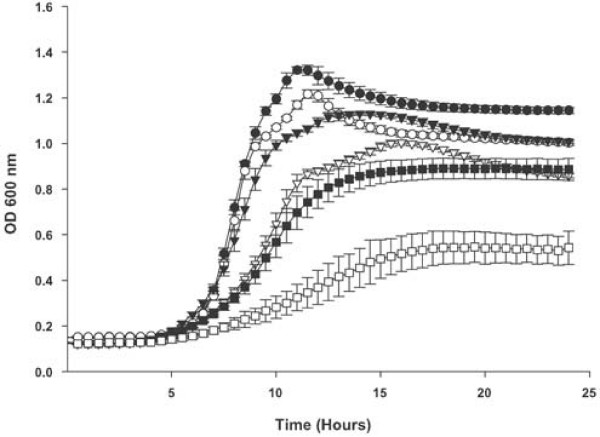
**Growth of *H. influenzae *strains with free heme as the sole heme source**. Growth of the *H. influenzae *strain Rd KW20 and the *dppC *insertion mutant strain TMV1262 in hdBHI supplemented with heme as the sole heme source. Wildtype strain Rd KW20 at 10 μg ml^-1 ^heme (solid circles), at 2 μg ml^-1^(solid triangles) and at 1.25 μg ml^-1 ^(solid squares). The *dppC *insertion mutant strain TMV1262 at 10 μg ml^-1 ^heme (open circles), at 2 μg ml^-1 ^(open triangles) and at 1.25 μg ml^-1 ^(open squares). Results are mean ± SD for quintuplicate results from representative experiments. The Kruskal-Wallis test was used to compare growth of Rd KW20 and TMV1262 over the entire 24 hour growth period at all heme concentrations (*P *< 0.0001 for all comparisons).

An insertional mutation in the *dppC *gene of *H. influenzae *strain Rd KW20 was constructed as part of an unrelated study [18]. The *dppC *mutant strain (TMV1262) contains a kanamycin resistance marker disrupting codon 290 of the *dppC*. The *dppC *mutant was compared to the wildtype strain in growth curve analyses for the ability to utilize various heme sources. Figure 1 shows comparisons of strains Rd KW20 and TMV1262 for utilization of free heme at 10, 2 and 1 μg ml^-1^, at all heme concentrations the *dppC *mutant strain grew significantly less well than the wildtype strain when compared over the entire growth period (*P *< 0.0001). In heme at 10 μg ml^-1 ^growth of the mutant strain was the same as the wildtype strain in the initial part of the growth curve through late exponential phase i.e. comparing growth over the first 10 hours *P *= 0.9882 and through 14 hours *P *= 0.1534. However, at the lower heme concentrations growth of the mutant was impaired compared to that of the wildtype strain from the onset of exponential phase. That growth in heme at 10 μg ml^-1 ^is only impacted at later time points likely reflects decreasing availability of heme. These data support the contention that the growth difference results specifically from a perturbation of heme acquisition rather than a general growth curve since in the latter circumstance the growth defect would be apparent at all heme concentrations.

**Figure 2:**
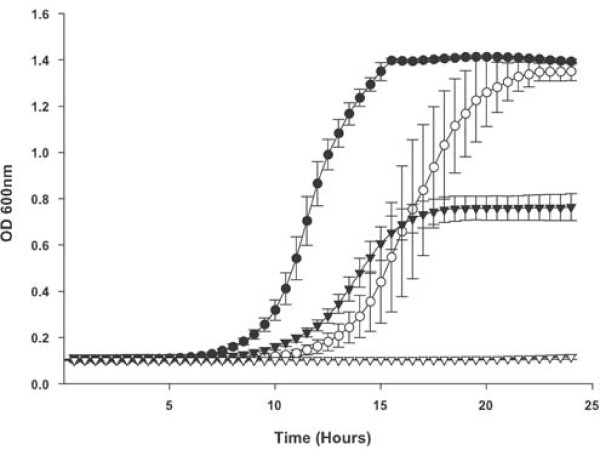
**Growth of *H. influenzae *strains with heme-human serum albumin as the sole heme source**. Growth of the *H. influenzae *strain Rd KW20 and the *dppC *insertion mutant strain TMV1262 in hdBHI supplemented with heme-human serum albumin as the sole heme source. Wildtype strain Rd KW20 with heme-human serum albumin at 200 ng ml^-1 ^heme equivalent (solid circles) and with heme-human serum albumin at 100 ng ml^-1 ^heme equivalent (solid triangles). The *dppC *insertion mutant strain TMV1262 with heme-human serum albumin at 200 ng ml^-1 ^heme equivalent (open circles) and with heme-human serum albumin at 100 ng ml^-1 ^heme equivalent (open triangles). Results are mean ± SD for quintuplicate results from representative experiments. The Kruskal-Wallis test was used to compare growth of Rd KW20 and TMV1262 over the entire 24 hour growth period at either concentration of heme-human serum albumin (*P *< 0.0001 for both comparisons).

**Figure 3:**
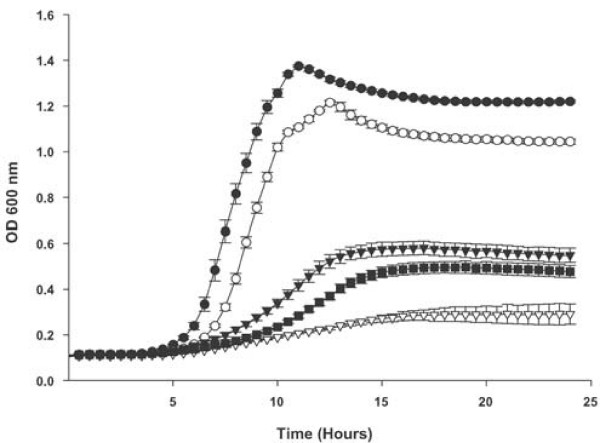
**Growth of *H. influenzae *strains with human hemoglobin as the sole heme source**. Growth of the *H. influenzae *strain Rd KW20, the *dppC *insertion mutant strain TMV1262 and the complemented *dppC *mutant strain HI2208 in hdBHI supplemented with human hemoglobin as the sole heme source. Wildtype strain Rd KW20 with human hemoglobin at 30 μg ml^-1 ^(solid circles) and at 10 μg ml^-1 ^(solid triangles). The *dppC *insertion mutant strain TMV1262 with human hemoglobin at 30 μg ml^-1 ^(open circles) and at 10 μg ml^-1 ^(open triangles). The complemented *dppC *mutant strain HI2208 with human hemoglobin at 10 μg ml^-1 ^(solid squares). Results are mean ± SD for quintuplicate results from representative experiments. The Kruskal-Wallis test was used to compare growth of Rd KW20 and TMV1262 over the entire 24 hour growth period at either concentration of hemoglobin *P *< 0.0001 and to compare growth of TMV1262 and HI2208 over the entire 24 hour growth period in 10 μg ml^-1 ^hemoglobin (*P *< 0.0001 for all comparisons).

**Figure 4:**
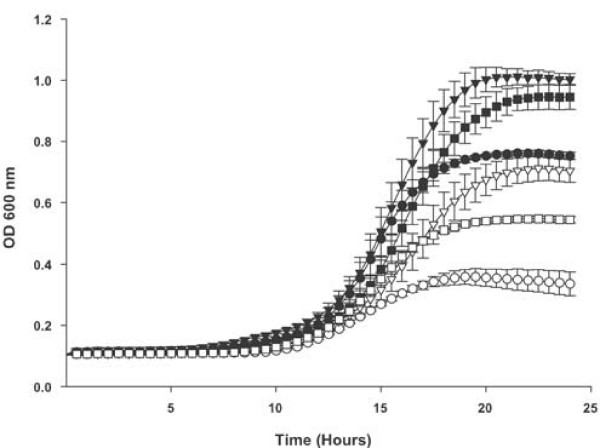
**Growth of *H. influenzae *strains with human hemoglobin as the sole heme source**. Growth of the *H. influenzae *strain Rd KW20, the *dppC *insertion mutant strain TMV1262 and the complemented *dppC *mutant strain HI2208 in hdBHI supplemented with human hemoglobin-haptoglobin complex as the sole heme source. Wildtype strain Rd KW20 with human hemoglobin-haptoglobin complex at 10 μg ml^-1 ^hemoglobin equivalent (solid triangles) and at 5 μg ml^-1^hemoglobin equivalent (solid circles). The *dppC *insertion mutant strain TMV1262 with human hemoglobin-haptoglobin complex at 10 μg ml^-1^hemoglobin equivalent (open triangles) and at 5 μg ml^-1 ^hemoglobin equivalent (open circles). The complemented *dppC *mutant strain HI2208 with human hemoglobin-haptoglobin complex at 10 μg ml^-1^hemoglobin equivalent (solid squares) and at 5 μg ml^-1 ^hemoglobin equivalent (open squares). Results are mean ± SD for quintuplicate results from representative experiments. The Kruskal-Wallis test was used to compare growth of Rd KW20 and TMV1262 or to compare growth of TMV1262 and HI2208 over the entire 24 hour growth period at either concentration of hemoglobin-haptoglobin (*P *< 0.0001 for all comparisons).

In addition to free heme the wildtype and *dppC *mutant strain were compared for utilization of various potential proteinaceous heme sources. In each case the *dppC *mutant strain grew significantly less well than the wildtype strain at all tested concentrations (Figure 2 for heme-human serum albumin; Figure 3 for hemoglobin; Figure 4 for hemoglobin-haptoglobin complexes). Onset of growth for the wildtype strain in both heme-human serum albumin and hemoglobin-haptoglobin complexes appears to be significantly delayed compared to that in either heme or hemoglobin. These observed differences may reflect the different concentrations used for each heme source. For example growth in heme was performed at 10, 2.5 and 1.25 μg ml^-1^, whereas growth in heme-human serum albumin complex was performed at 200 or 100 ng ml^-1 ^with respect to heme. These apparent differences in onset of growth may also reflect different affinities of the appropriate *H. influenzae *outer membrane receptor proteins for their substrate, although no data is currently available to support this contention.

**Figure 5:**
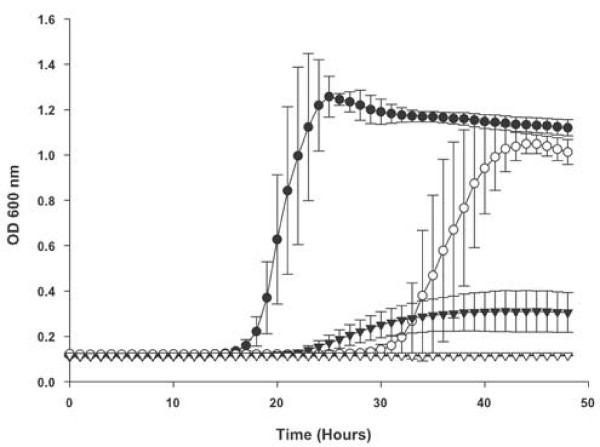
**Growth of *H. influenzae *strains with protoporphyrin IX**. Growth of the *H. influenzae *strain Rd KW20 and the *dppC *insertion mutant strain TMV1262 in hdBHI supplemented with protoporphyrin IX at either 2.5 or 0.5 μg ml^-1 ^and additionally supplemented with 200 μM ferrous ammonium sulfate. Wildtype strain Rd KW20 with protoporphyrin IX at 2.5 μg ml^-1 ^(solid circles) and at 0.5 μg ml^-1 ^(open circles). The *dppC *insertion mutant strain TMV1262 with protoporphyrin IX at 2.5 μg ml^-1 ^(closed triangles) and at 0.5 μg ml^-1 ^hemoglobin equivalent (open triangles). Results are mean ± SD for quintuplicate results from representative experiments. The Kruskal-Wallis test was used to compare growth of Rd KW20 and TMV1262 over the entire 48 hour growth period at either concentration of protoporphyrin IX (*P *< 0.0001 for both comparisons). Optical density measurements were taken every 30 minutes, however, for the purpose of the clarity of data presentation only hourly time points have been plotted.

In addition to the above studies with various heme sources the impact of the *dppC *mutation on the utilization of PPIX was also determined. Growth curves were performed with hdBHI supplemented with PPIX at various concentrations in the presence of 200 μM ferrous ammonium sulfate. Figure 5 shows comparisons of strains Rd KW20 and TMV1262 for utilization of PPIX at 2.5 and 0.5 μg ml^-1^, at both concentrations of PPIX growth of the dppC mutant strain was significantly impaired compared to that of the wildtype strain (*P *< 0.0001 when comparisons were made over the entire growth period).

That the growth defects reported in this manuscript result from mutation of *dppC *is supported by the observation that complementation of the mutant strain with an intact *dppBCDF *gene cluster corrected the growth defect reported for the mutant strain (data is shown for growth in hemoglobin at 10 μg ml^-1 ^in Figure 3 and in hemoglobin-haptoglobin at both 10 and 5 μg ml^-1 ^in Figure 4).

The data reported herein indicate that the *dppBCDF *operon constitutes part of the *H. influenzae *periplasmic heme/porphyrin transport system(s). However, since heme utilization is not completely abrogated, it is clear that an additional periplasmic system(s) must be available to transport heme. Several potential candidates for such a system(s) have been identified [9]. One additional locus potentially involved in periplasmic heme transport is the *sap *operon. The *sap *operon comprises the genes SapABCDFZ (HI1638-HI1643 in strain Rd KW20) and is involved in resistance to antimicrobial peptides [22]. The SapABCDF proteins show significant homology to HbpA and the *H. influenzae *DppBCDF proteins, and preliminary studies indicate a potential role for the *sap *operon in heme utilization [23]. Two additional putative periplasmic proteins are homologous to both HbpA and SapA and may be involved in heme acquisition; these two proteins are encoded by the ORFs designated HI0213 and HI1124 in the *H. influenzae *strain Rd KW20 genomic sequence [14]. In a microarray study of the response of Rd KW20 to iron and heme levels in the growth media the ORF HI0213 was maximally transcribed under conditions of iron/heme restriction, supporting a potential role in heme acquisition [24], although in two additional strains HI0213 transcript levels were not affected by iron/heme levels [25]. In Rd KW20 the locus HI0213 is a stand alone gene encoding a putative permease component of an ABC transporter, which could potentially interact with the DppBCDF proteins. The locus HI1124 is the permease component of an ABC transporter encoded by the operon HI1120-HI1124 and designated OppABCDF in the original Rd KW20 sequencing project [14]. Although there is as yet no empirical data for a role of either HI0213 or OppABCDF in heme utilization based on homology to HbpA and DppBCDF they warrant further investigation.

In conclusion a role for the *dppBCDF *locus of *H. influenzae *in periplasmic heme/porphyrin transport has been identified. Further studies will seek to elucidate additional periplasmic heme/porphyrin transport systems, and clarify the precise roles of HbpA and DppBCDF.

## Competing interests

The authors declare that they have no competing interests.

## Authors' contributions

All authors contributed to the design and execution of the experiments detailed. TMV constructed the *dppC *mutant strain. DJM constructed the complementation vector and performed growth studies. DJM drafted the manuscript. PWW, TWS and TLS revised the manuscript. All authors read and approved the final manuscript.
